# Low-dose epidural morphine for postpartum pain relief: a randomized, single-blind study

**DOI:** 10.1186/s40981-025-00818-4

**Published:** 2025-09-26

**Authors:** Hiroaki Kondo, Shunsuke Hyuga, Yoshinori Tomoda, Tomoe Fujita, Mariko Adachi, Toshiyuki Okutomi

**Affiliations:** 1 Division of Obstetric Anesthesia, Center for Perinatal Care, Child Health and Development, Kitasato University Hospital, 1-15-1 Kitasato Minami-Ku, Sagamihara City, Kanagawa, 252-0375 Japan; 2 Laboratory of Clinical Pharmacokinetics, Research and Education Center for Clinical Pharmacy, Kitasato University School of Pharmacy, 5-9-L Shirokane, Minato-Ku, Tokyo, 108-8641 Japan

**Keywords:** Postpartum pain relief, Labor analgesia, Epidural morphine

## Abstract

**Introduction:**

Epidural morphine administration following vaginal delivery reduces postpartum pain; however, side effects may occur. We investigated whether a lower dose could achieve pain relief without increasing the incidence and severity of side effects.

**Methods:**

Eighty women treated with combined spinal-epidural analgesia received 0.75 mg epidural morphine (morphine group) or normal saline (normal saline group) after delivery. The primary outcome was the area under the curve (AUC) of the visual analog scale score assessing perineal and contraction pain for 24 h following delivery. Secondary outcomes were time until initial request for additional analgesics, number of analgesic medications, and side effects incidence and severity.

**Results:**

The morphine group did not show lower mean AUCs for postpartum perineal (290, interquartile range [IQR]: 90–580 vs 450, IQR: 265.6–760;* P* = 0.07) or contraction pain (18.8, IQR: 0–105 vs 156.3, IQR: 11.5–300; *P* = 0.004). The time until the initial request for additional analgesics was longer in the morphine group (530 min, 95% confidence interval [CI]: 365 to 915 min vs 268 min, 95% CI: 230 to 385 min; *P* = 0.001). The median (IQR) number of analgesic medications within 24 h were 2 (0.5–3) and 2.5 (2–3) in the morphine and saline groups, respectively (*P* = 0.007). There were no differences in the incidence of side effects between the groups.

**Conclusions:**

Epidural morphine following vaginal delivery reduces contraction pain but not perineal pain and prolongs the time until initial request for additional analgesia without increasing side effects.

**Clinical trials registration number:**

The University Hospital Medical Information Network Clinical Trials Registry (registration number: UMIN000039351).

## Background

Childbirth is one of the most painful events that a woman can experience in her lifetime [1]. Vaginal delivery may lead to significant pain during the postpartum period, which is caused by episiotomy, perineal tears, and pain due to uterine contraction after vaginal delivery [2]. It has been reported that many pregnant women experience postpartum pain [3–5]. In addition, inadequate postpartum analgesia after vaginal delivery can impair the bonding between mother and newborn, disrupt lactation, and be severe enough to interfere with the care of the infant [6]. A study on the prevention of lacerations has been conducted [7], and further research exploring the valuable role of anesthesiologists in preventing severe perineal lacerations and managing uterine contraction pain after vaginal delivery would be highly beneficial [8, 9]. Therefore, all women should be considered eligible for analgesic strategies after vaginal delivery in the immediate postpartum period [2].

It has been suggested that the postpartum administration of epidural morphine may be beneficial for women who have received epidural labor analgesia during vaginal delivery [10]. Previous studies have demonstrated that the dosage of epidural analgesia varies (1–4 mg) [2, 10, 11]. The administration of 1–2 mg of epidural morphine has been reported to reduce the requirement for oral pain medication during the initial 24 h postpartum [2]. Furthermore, a study reported no significant difference in the side effects during the first 24 h between patients administered 2.5 mg of epidural morphine and those given saline placebo [10]. Another study reported that while 2 mg of epidural morphine was effective for analgesia, increasing the dose to 4 mg did not enhance the analgesic efficacy but was associated with more side effects [11].

Although morphine is an effective analgesic, its side effects may negatively impact the quality of life after childbirth. Therefore, we sought to identify a regimen that maintains the analgesic effect while minimizing side effects. Herein, we aimed to explore whether the administration of morphine at a lower dose (0.75 mg) than previously reported in other studies could achieve postpartum pain relief without increasing the incidence and severity of side effects.

## Methods

### Aim

We aimed to explore whether the administration of morphine at a lower dose (0.75 mg) than previously reported could achieve postpartum pain relief without increasing the incidence and severity of side effects.

### Study design and setting

This single-center, randomized, single-blind clinical trial was approved by the Kitasato University Medical Ethics Organization (C19-300) on February 12, 2020 and was conducted at the Kitasato University Hospital, Kanagawa, Japan, from November 2020 to May 2022. This trial was registered prior to patient enrollment in the University Hospital Medical Information Network Clinical Trials Registry (registration number: UMIN000039351). The manuscript adheres to the Consolidated Standards of Reporting Trials (CONSORT) guidelines [12]. Prior to the start of the first stage of labor, patients provided written informed consent for participation in this study.

### Participants

The study included patients who underwent vaginal delivery; received an effective Combined Spinal Epidural Analgesia (CSEA) for labor and delivery; nulliparous or primiparous; with an American Society of Anesthesiologists physical status score of 2; aged ≥ 20 years; and a single gestation in the vertex presentation position at term. The exclusion criteria included: a history of gestational diabetes mellitus or chronic pain syndrome; a history of drug abuse; and chronic use of pain medications, antidepressants, or anticonvulsants. The following data were recorded: parity; maternal age and body mass index; gestational age; neonatal birth weight; the volume of local anesthetic used for breakthrough pain, episiotomy, and/or degree of perineal laceration; spontaneous delivery or operative vaginal delivery; and whether vacuum or forceps were used. The perineal laceration was defined as minor or major, with minor laceration indicating no, periurethral, or first-degree laceration, and major laceration indicating second-, third-, or fourth-degree laceration [13].

### Randomization and blinding

A random number generator was used to select random permuted blocks with a block size of four, stratified for primiparous and multiparous participants. Random allotment was performed using a computer-generated random table and the sealed enveloped technique by an independent anesthesiologist. The patients were blinded to conceal group allocation and therefore could not influence the data.

### Procedure for labor and delivery analgesia

Labor analgesia was performed using the needle-through-needle CSEA technique at L3–L4 with an 18G Tuohy needle, loss of resistance to saline or air, and a 27G pencil-point spinal needle (Espocan®B-Braun, Melsungen, Germany). The administration of spinal analgesia used 2.0 mg of isobaric bupivacaine and 20 µg of fentanyl. A multi-orifice epidural catheter was then inserted 4 cm into the epidural space. Epidural analgesia was maintained with 0.08% levobupivacaine and 2 µg/mL of fentanyl. Settings for the programmed intermittent epidural bolus (PIEB) pump (CADD®-Solis, Smiths Medical, St Paul, MN, USA) included a background infusion of 3 mL/h and a programmed bolus of 6 mL/h. Thirty min after the spinal injection, the pump was started and PIEB analgesia was initiated with a 6 mL bolus. It was maintained with a programmed bolus of 6 mL/h. Breakthrough pain was defined as maternal complaints of pain or painful pressure that required one or more doses of unplanned epidural medication [14]. The choice of medications for supplementation was the responsibility of the anesthesiologist in charge of the patient's care. The infusion was continued until the third stage of labor was completed.

### Procedures for epidural administration of morphine or normal saline after delivery

Within 60 min of delivery, patients received a 1 ml epidural injection containing 0.75 mg morphine or saline. The epidural catheter was removed prior to patients being transferred to the postpartum ward.

### Assessment of postpartum pain

A standard anesthesia order set with monitoring guidelines, side effects management, and analgesia management was used for the first 24 h following discharge to the postpartum ward. Nursing staff monitored the patients at 1, 2, 6, 12, 18, and 24 h postpartum for respiratory rate, nausea/vomiting, and pruritus. The visual analog scale (VAS) pain score was obtained separately for perineal and postpartum contraction pain on a 0–100 mm scale. Additional analgesics, including oral nonsteroidal anti-inflammatory drugs (NSAIDs), specifically loxoprofen sodium (60 mg every 6 h as needed) or rectal diclofenac suppositories (25 mg every 6 h), could be requested for breakthrough perineal and postpartum contraction pain. The choice of supplemental analgesics was made in cooperation with patients and nurses under the order of a physician.

### Assessment of side effects (nausea, pruritus, urinary retention, and respiratory depression)

The patients were monitored at 1, 2, 6, 12, 18, and 24 h postpartum by the nursing staff for nausea/vomiting, pruritus, and respiratory rate. Side effect incidence and severity were assessed. Nausea and pruritus severity were measured using a 4-point ordinal verbal rating scale (0 = none; 1 = mild; 2 = moderate; and 3 = severe) [15]. Requirement of a catheter or intermittent catheterization 6 h postpartum defined urinary retention. Respiratory depression was defined as a respiratory rate of < 10 breaths/min.

### Primary and secondary outcomes

The primary outcome was a comparison of pain for the first 24 h postpartum, which was separately measured for perineal and postpartum contraction pain. We compared the VAS-area under the curve (AUC) using linear extrapolation between the time points to calculate AUC for pain measurements. We defined the primary outcome as being achieved if there was a statistically significant difference for both perineal pain and uterine contraction pain. We conducted a sensitivity analysis on pregnant women who delivered in this study, excluding those who had received analgesic medication more than twice within a 24 h period. We then compared postpartum perineal and contraction pain between the two groups. Based on a previous study [2], pregnant women who used analgesics more than twice during 24 h were excluded. The secondary outcomes were the time until the initial request for additional analgesics (starting from the epidural administration and censored at 24 h after the procedure), the number of analgesic medications administered during the 24 h period, and the incidence and severity of side effects (nausea, pruritus, urinary retention, and respiratory depression).

### Statistical analysis

All data were analyzed on an intent-to-treat basis. Adequate information could not be retrieved from previous literature regarding perineal and postpartum contraction pain based on the VAS-AUC measurements to calculate power. Therefore, the mean and standard deviation (SD) of VAS measurements were used assuming a proportional relationship between the mean VAS and VAS-AUC [16]. The sample size was calculated based on the study by Dannecker et al. [17], who reported an at-rest mean (SD) pain score following liberal use of mediolateral episiotomy and/or perineal trauma (1–5 days post-episiotomy) of 39 ± 28 mm using a VAS ranging from 0–100 mm. They determined that 36 women were required for each group (5% level of significance, two-sided, with 80% power) in order to detect significantly reduced pain scores of 20 mm (with a similar SD) for mean VAS (corresponding to VAS-AUC of 480 VAS score units × hours). We therefore included 40 women in each group. Postpartum pain over time (VAS score) was plotted for the two groups, and the AUC was estimated for each patient. Based on a previous study, [17] we assumed that the mean VAS-AUC is normally distributed. The Kolmogorov–Smirnov test was used to verify the normal distribution of the mean VAS-AUC. The means for the VAS-AUC of the two groups were not normally distributed. The mean VAS-AUCs were compared according to the randomization arm using the Mann–Whitney U test. To address missing VAS date, we used multiple imputation with M = 100 imputations, following Rubin’s framework [18]. The time until the initial request for additional analgesics was compared using the log-rank test. The number of analgesic medications administered during the 24 h period was compared using the Mann–Whitney U test. Side effects (nausea, pruritus, urinary retention, and respiratory depression) were analyzed using Fisher’s exact test (two-sided), wherein the side effects scales used a linear mixed-effect model. The fixed effects were the hour, drug, and hour × drug interaction, and the random effects were for the study participants. Statistical significance was set at *P* < 0.05, and hour × drug interaction was defined as *P* < 0.10 to indicate a potentially relevant interaction. EZR was used to perform statistical analyses (Saitama Medical Center, Jichi Medical University, Saitama, Japan) [19] and is a graphical user interface for R (The R Foundation for Statistical Computing, Vienna, Austria) and R Commander.

## Results

**Fig. 1:**
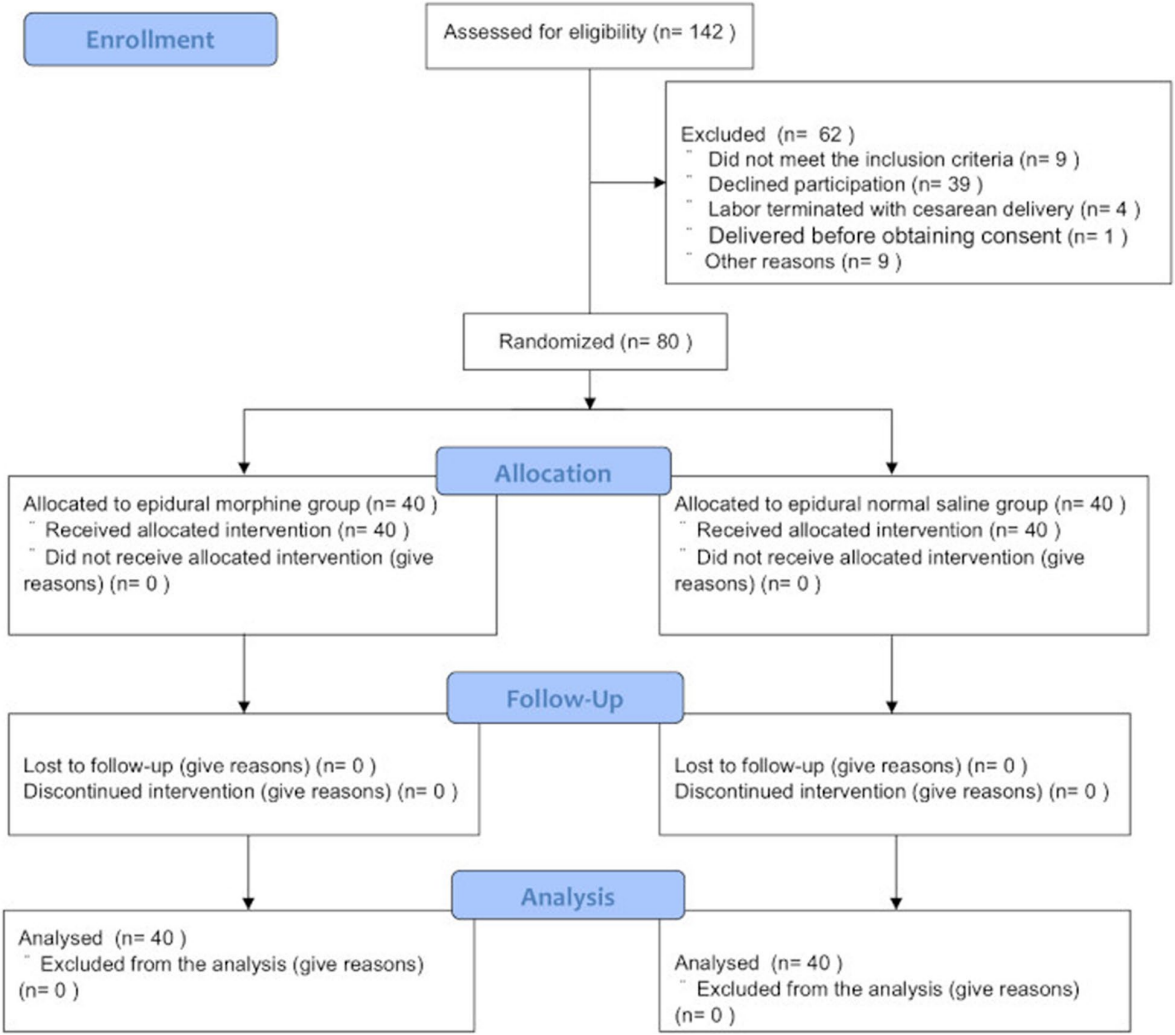
CONSORT flow diagram. CONSORT recommended recruitment of patients to the single-blind, placebo-controlled trial, who were randomized to receive morphine or saline. The study was conducted at the Kitasato University Hospital, Kanagawa, Japan

Among 142 women assessed for eligibility, 80 were randomly allocated to the epidural morphine hydrochloride or normal saline groups (Fig. 1). Forty participants per group completed the allocated intervention and were included in the final analysis. Patient baseline characteristics and intraoperative details are summarized in Table 1.

**Table 1:** Demographic and obstetric data

	Morphine group *N* = 40	Saline group *N* = 40	*P* value	Standardized difference
Primiparous, *n*/total* N* (%)	20/40 (50%)	20/40 (50%)	1.00	0
Maternal age (yr), mean (SD)	34 (4.2)	34 (5.2)	0.65	0.10
Maternal BMI (kg/m^2^), mean (SD)	25 (3.3)	24 (2.5)	0.72	0.08
Gestational age (weeks), mean (SD)	38 (0.8)	38 (0.8)	0.77	0.07
Neonatal birth weight (g), mean (SD)	2,967 (342)	2,961 (369)	0.93	0.02
Volume of local anesthetic for breakthrough pain(ml), median (IQR)	4.0 [1.5–8.0]	5.5 [3.0–9.9]	0.17	0.20
Perineal trauma				
Episiotomy, *n*/total* N* (%)	31/40 (77%)	29/40 (73%)	0.80	0.12
First degree or less, *n*/total* N* (%)	17/40 (43%)	18/40 (45%)	1.00	0.05
Second degree or greater, *n*/total* N* (%)	23/40 (58%)	22/40 (55%)	1.00	0.05
Operative vaginal delivery, *n*/total* N* (%)	18/40 (45%)	23/40 (58%)	0.37	0.25

_*BMI *Body mass index, *SD *Standard deviation, *IQR *Interquartile range_

### Postpartum pain assessment

**Fig. 2:**
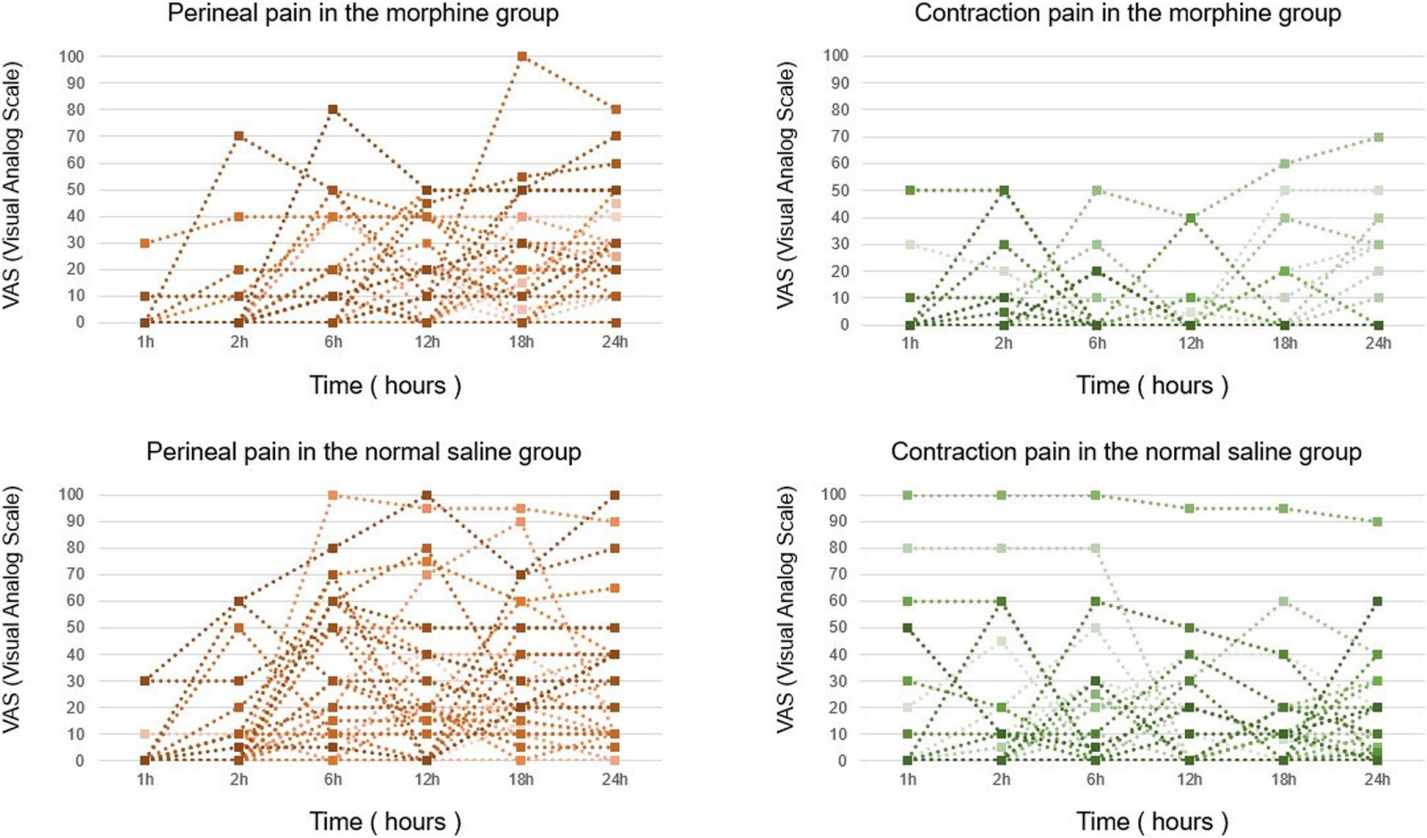
Serial VAS scores for perineal pain and contraction pain after vaginal delivery in the morphine and normal saline groups

**Fig. 3:**
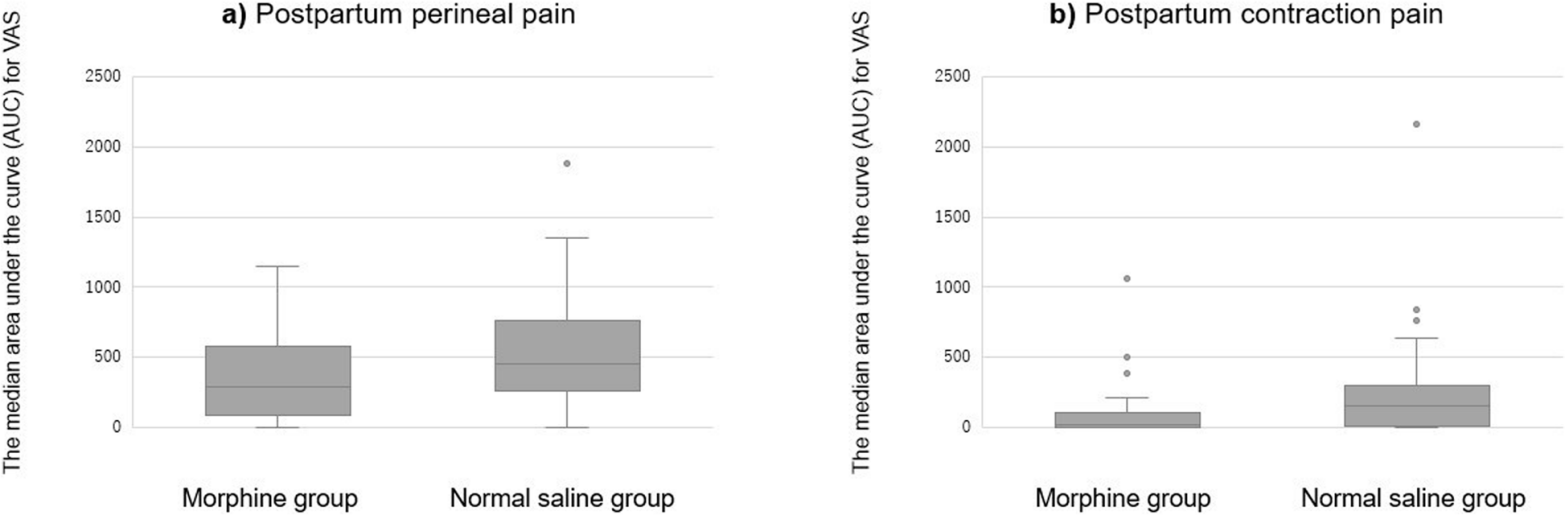
The median area under the curve (AUC) for VAS in the two groups. The median VAS-AUC for (**a**) perineal pain and (**b**) contraction pain. The box plot shows the median (the solid line), 25th and 75th percentiles (lower and upper limits of the box), and the minimum and maximum observations (whiskers). AUC, area under the curve; IQR, interquartile range; VAS, visual analog scale

**Fig. 4:**
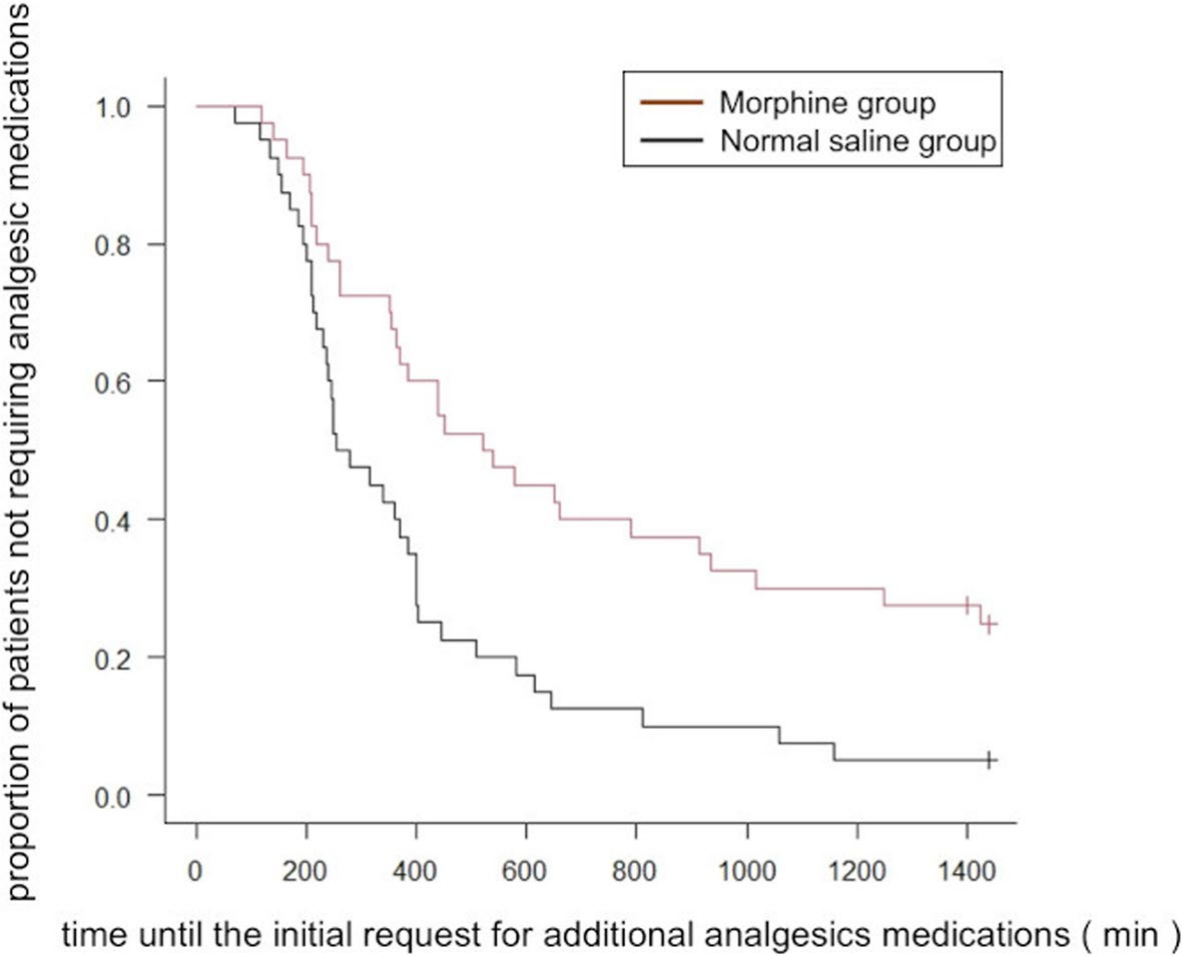
Kaplan–Meier curves analyzing the time until the initial request for additional analgesia in both groups. The time until the initial request for additional analgesics was significantly longer in the morphine group than the normal saline group (*P* = 0.001 by the log-rank test)

Figure 2 illustrates the time courses of postpartum perineal pain and contraction pain, as assessed by VAS at each time point. The median (interquartile range [IQR]) AUCs for postpartum perineal pain were 290 (90–580) and 450 (265.6–760) VAS score units × hours in the morphine and normal saline groups, respectively (*P* = 0.07) (Fig. 3a). For postpartum contraction pain, the median (IQR) AUCs were 18.8 (0–105) and 156.3 (11.5–300) VAS score units × hours in the morphine and normal saline groups, respectively (*P* = 0.004) (Fig. 3b). The sensitivity analysis yielded the following results: postpartum perineal pain and uterine contraction pain were compared in 56 women after excluding 24 women who required analgesics two or more times within the first 24 h after delivery. The median (IQR) AUCs for postpartum perineal pain were 435 (202.5–766.3) and 525 (313.8–850) VAS score units × hours in the morphine and normal saline groups, respectively (*P* = 0.214). For postpartum contraction pain, the median (IQR) AUCs were 0 (0–70) and 180 (36.8–300) VAS score units × hours in the morphine and normal saline groups, respectively (*P* = 0.0014). The median time until the initial request for additional analgesics was 530 min (95% confidence interval [CI]: 365–915) and 268 min (95% CI: 230 to 385) in the morphine and normal saline groups, respectively. The duration was significantly prolonged in the morphine group (*P* = 0.001 using the log-rank test; Fig. 4). was 2 (0.5–3) and 2.5 (2–3) in the morphine and normal saline groups, respectively. umber of administered analgesic medications was lower in the morphine group than in the normal saline group (*P* = 0.007). Some of the data corresponding to VAS scores of perineal and uterine contraction pain at 1 h after epidural administration of normal saline was missing (in 2 out of 40 patients from the control group).

### Side effects

**Fig. 5:**
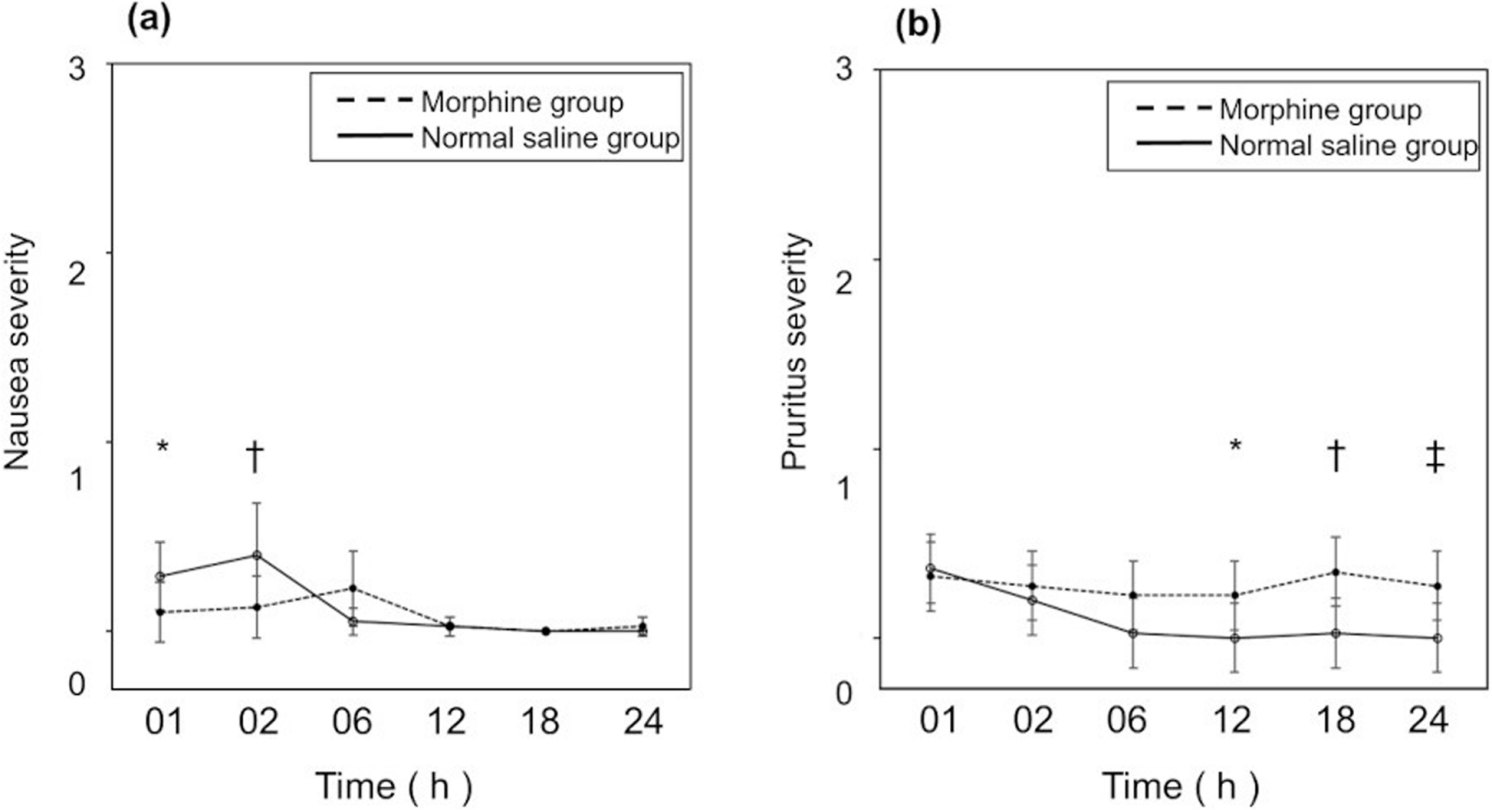
Severity of nausea and pruritus throughout the study. Nausea (**a**) and pruritus (**b**) severity were assessed using a 4-point verbal rating scale (0 = none; 1 = mild; 2 = moderate; 3 = severe) [13]. Linear mixed-effects modeling showed that nausea severity decreased over time, with significant group differences at 1 h and 2 h (*P* = 0.04, † *P* = 0.003). Pruritus severity was lower in the saline group than in the morphine group at 12, 18, and 24 h (* *P* = 0.04, † *P* = 0.003, ‡ *P* = 0.01). Whiskers indicate 95% confidence intervals

The incidence of nausea, including mild, moderate, and severe, was 23% (9/40) and 28% (11/40) in the morphine and normal saline groups, respectively; no statistically significant difference was observed between the two groups (odds ratio [OR] = 0.77, 95% CI: 0.24–2.38, *P* = 0.79; Table 2). For nausea, the hour × drug interaction was significant (*P* = 0.005). A linear mixed-effects model for exploratory analysis showed a decrease in nausea severity over time. Significant differences were observed between the groups at 1 h (*P* = 0.04) and 2 h (*P* = 0.003; Fig. 5a), with the normal saline group reporting higher severity of nausea compared with morphine group. The incidence of pruritus, including mild, moderate, and severe, was 58% (23/40) and 40% (16/40) in the morphine and normal saline groups, respectively; no statistically significant difference was observed between the two groups (OR = 2.01, 95% CI: 0.76 to 5.4, *P* = 0.18; Table 2). For pruritus, the hour × drug interaction was significant (*P* = 0.06). The analysis further showed a lower incidence of pruritus in the normal saline group than in the morphine group at 12 h (*P* = 0.04), 18 h (*P* = 0.003), and 24 h (*P* = 0.01; Fig. 5b). The incidence of urinary retention was 48% (19/40) and 28% (11/40) in the morphine and normal saline groups, respectively; no statistically significant difference was observed in the need for urinary catheter or intermittent catheterization 6 h postpartum (OR = 2.36, 95% CI: 0.86 to 6.7, *P* = 0.11; Table 2). None of the women required urinary catheterization after 24 h. No cases of respiratory depression were observed (Table 2).

**Table 2:** Incidence of side effects

	Morphine group *N* = 40	Saline group *N* = 40	Odds ratio (95% CI)	*P* value
Nausea, *n*/total* N* (%)	9/40 (23%)	11/40 (28%)	0.77 (0.24 to 2.38)	0.79
Pruritus, *n*/total* N* (%)	23/40 (58%)	16/40 (40%)	2.01 (0.76 to 5.4)	0.18
Urinary retention, *n*/total *N* (%)	19/40 (48%)	11/40 (28%)	2.36 (0.86 to 6.7)	0.11
Respiratory depression, *n*/total* N* (%)	0/40 (0%)	0/40 (0%)	–	–

^Numbers are presented as proportions (%) of both groups who experienced side effects. The odds ratio was calculated for the normal saline group relative to the morphine group^

^*CI*^ ^Confidence interval^

^*P*^ ^values from Fisher’s exact test^

## Discussion

The present study showed a significant difference in VAS-AUC for contraction pain between the normal saline and morphine groups, but not significant difference was observed for perineal pain. As achievement of the primary outcome was defined as the presence of statistically significant differences in both perineal and contraction pain, this single-blind, randomized controlled trial failed to achieve this outcome. The time until the initial request for additional analgesics was significantly longer in the morphine group than in the normal saline group. The median number of analgesic medications administered in the first 24 h was lower in the morphine group than in the normal saline group. No significant difference in the incidence of side effects was observed between the groups.

Other randomized trials have evaluated the use of epidural morphine for post-vaginal delivery pain [2, 10, 11] wherein women were randomized to receive 1–4 mg of either epidural morphine or saline. We chose a 0.75 mg epidural morphine dose for our study based on the absence of studies that compared the effect of < 1 mg of morphine. The difference in VAS-AUC for the primary outcome, that is, postpartum perineal and contraction pain, between the morphine and normal saline groups was not statistically significant. A potential explanation for this outcome is that we did not restrict the use of additional analgesics. However, the sensitivity analyses ruled out the possibility that additional analgesics may have masked the effect of morphine. Therefore, it is possible that the analgesic effect of morphine was minimal, or that the dose was inadequate. Although no statistical difference in VAS-AUCs was observed in this study, the normal saline group required more additional analgesics than the morphine group. Furthermore, the time until the initial request for additional analgesics was significantly longer in the morphine group than in the normal saline group. This may suggest that epidural morphine provided long-term analgesia rather than peak pain suppression.

The episiotomy rates in this trial were high in both the morphine (77%) and saline (73%) groups. Dannecker et al. reported that the liberal use of episiotomies was associated with increased perineal pain [17]. Thus, the women in our study may be considered a high-risk group, especially for increased perineal pain. While episiotomy may have influenced the primary outcome of this study, whether this completely masked the effect of morphine remains unclear. Future research should investigate the type of episiotomy performed and its relationship with the pain experienced. In addition, previous studies have reported that severe uterine contraction and perineal pain after delivery can prevent appropriate resting and cause anxiety or depression [20]. Therefore, further research is essential to investigate the long-term effects of perineal pain and uterine contraction pain in pregnant women who received epidural morphine.

In this study, analgesics were used for postpartum pain at the patient’s request. However, few studies have focused on the implementation of a standardized analgesic regimen in women who have undergone vaginal delivery [21]. In contrast, for cesarean delivery, scheduled administration of analgesics, combined with intrathecal morphine, has been shown to be effective in postoperative pain management [22]. A similar multimodal analgesic approach, incorporating low-dose epidural morphine, may also be beneficial for women after vaginal delivery. In addition, the cervix and vagina receive visceral afferent projections via both the hypogastric and pelvic nerves [23]. Consequently, perineal pain resulting from episiotomy or vaginal laceration likely involves not only somatic but also visceral components. Morphine exerts analgesic effects by directly inhibiting nociceptive transmission at both the dorsal horn of the spinal cord and peripheral nociceptive afferent neurons [24], making it particularly effective for visceral pain. Given that postpartum pain involves a complex combination of somatic and visceral pain, the addition of low-dose epidural morphine to basic analgesia with NSAIDs and acetaminophen may be a valuable strategy for optimizing postpartum pain management. In the future, there is a need to adopt a standardized analgesic regimen and to evaluate the effects of administering relatively small doses of epidural morphine.

This study found no significant differences in the incidence of nausea, pruritus, or urinary retention between the two groups. Previously, only the incidence of side effects (nausea and pruritus) had been assessed [10], whereas our study used a 4-point ordinal verbal rating scale. Urinary retention has been previously defined as the need for catheterization (without any specific definitions for timeframes) [10], whereas it was defined as the need for a catheter or intermittent catheterization requirements 6 h after delivery in the present study. Thus, despite a more step-wise assessment of even minor side effects compared to previous studies, the morphine group did not experience more side effects compared with the normal saline group. The planned exploratory analysis demonstrated a decrease in the severity of nausea over time and a lower degree of pruritus in both groups. Importantly, our study assessed the severity of nausea and pruritus over time, which was low in both groups at each time point during the study period. None of the women required urinary catheterization after 24 h. Since all patients should remain hospitalized for at least 24 h after vaginal delivery for observation [25], a relatively lower dose of epidural morphine (0.75 mg) can be safely used while assessing the incidence and severity of side effects such as nausea, pruritus, urinary retention, and respiratory depression.

This study has some limitations. First, this was a single-blinded study; because of institutional reasons, our institutional review board did not provide approval for a double-blinded design. There were concerns that not informing the nursing staff about the intervention could become a medical safety issue. Second, this study had a small randomization of four blocks. Small blocks could make blinding difficult. Randomization in larger blocks may have been more appropriate [26]. Third, additional analgesics were added as required for perineal or postpartum contraction pain. Therefore, we were unable to standardize a regimen for scheduled analgesic medication. Fourth, although the proportion of episiotomy did not differ between the two groups, women who underwent episiotomy are expected to experience greater perineal pain. Therefore, episiotomy may still have influenced individual pain trajectories. Future studies may need to stratify participants according to the presence or absence of episiotomy to more precisely evaluate postpartum pain outcomes. Finally, this study did not directly compare the effects of 0.75 mg epidural morphine with those of 1–2 mg epidural morphine, and therefore no conclusions can be drawn regarding the superiority of either dose. Further dose–response studies are required to determine the optimal dose.

## Conclusions

Low-dose epidural morphine reduced analgesic use during the initial 24 h postdelivery without increasing side effects, but no change in postpartum analgesia was observed between the groups. Further research is required to determine the optimal dose with fewer side effects and higher analgesic effects.

## Data Availability

The corresponding author will provide the data upon reasonable request.

## Abbreviations

AUC: Area under the curve
CI: Confidence interval
CSEA: Combined Spinal Epidural Analgesia
IQR: Interquartile range
NSAIDs: Nonsteroidal anti-inflammatory drugs
PIEB: Programmed intermittent epidural bolus
SD: Standard deviation
VAS: Visual analog scale
